# *Babesia divergens* in human in Gansu province, China

**DOI:** 10.1080/22221751.2019.1635431

**Published:** 2019-06-27

**Authors:** Jinming Wang, Shangdi Zhang, Jingqiong Yang, Junlong Liu, Dekui Zhang, Youquan Li, Jianxun Luo, Guiquan Guan, Hong Yin

**Affiliations:** a State Key Laboratory of Veterinary Etiological Biology, Key Laboratory of Veterinary Parasitology of Gansu Province, Lanzhou Veterinary Research Institute, Chinese Academy of Agricultural Sciences, Lanzhou, People’s Republic of China; b Department of Clinical Laboratory, The Second Hospital of Lanzhou University, Lanzhou, People’s Republic of China; c Department of Clinical Laboratory, The Hospital of Gannan Tibetan Medicine, Gannan Tibetan Autonomous Prefecture, People’s Republic of China; d Department of Gastroenterology, The Second Hospital of Lanzhou University, Lanzhou, People’s Republic of China; e Jiangsu Co-Innovation Center for the Prevention and Control of Important Animal Infectious Disease and Zoonose, Yangzhou University, Yangzhou, People’s Republic of China

**Keywords:** Human babesiosis, Babesia divergens, China, Gansu, prevalence

## Abstract

Human babesiosis is an important tick-borne infectious disease. We investigated human babesiosis in the Gansu province and found that it is prevalent in this area with a prevalence of 1.3%. Results of gene sequencings indicate that 1.3% of patients were positive for *Babesia divergens*. This initial report of human *B. divergens* infections in Gansu Province should raise awareness of human babesiosis.

Human babesiosis, caused by parasites of the genus *Babesia*, is an important tick-borne infectious disease that is predominantly transmitted by tick bites and by infected blood transfusions [1]. The first case of human babesiosis was reported in Zagreb, Croatia, in 1957 and subsequently in Asia, Africa, South and North America, and Europe [1–3]. At least nine *Babesia* spp. have been identified so far from humans: *Babesia crassa*, *Babesia* sp. TW1, and *Babesia* sp. XXB/HangZhou (China), *Babesia microti* (China, Europe, Bolivia and America) [3–5], *Babesia divergens* and *Babesia* sp. EU1/*Babesia venatorum* (China and Europe), *Babesia microti*-like (Japan), *Babesia duncani* (America) and *Babesia* sp. KO1 (Korea) [6–10].

Recently, increasing numbers of cases of human babesiosis have been reported in China, caused by two new emerging *Babesia* species, named *B. crassa* and *Babesia* sp. XXB/HangZhou. However, few surveys of the prevalence of human babesiosis have been conducted in northeastern China. Available results revealed the higher prevalence of *B. crassa* and *B. venatorum* [11]. However, despite the wide distribution of tick species in Gansu province, northwestern China, including 7 *Ixodes* spp., 13 *Haemaphysalis* spp., 5 *Hyalomma* spp. and 3 *Dermacentor* spp. which are all possible vectors of *Babesia* spp. that are infective to humans, no cases or relevant epidemiological data have been published.

This study was conducted in a total of 754 patients who lived in the Gannan Tibetan Autonomous Prefecture, Gansu province and visited the Second Hospital of Lanzhou University for a tick bite in the past few months between April 2016 and March 2018. A standardized questionnaire was applied to record the patient's basic information, including sex, age, career, and history of tick bites and blood transfusion. Two blood samples were taken from each patient: one was sent to the medical laboratory for clinical examination, whereas the second was analysed using nested PCR (nPCR) assays to detect the presence of any *Babesia* spp. All participants agreed to participate in this study and signed an informed consent form. The study was approved by the Ethics Committee of The Second Hospital of Lanzhou University (reference 2018A-046). All the procedures conducted were according to the Ethical Procedures and Guidelines of the People's Republic of China.

The collected blood specimens were used to extract genomic DNA by using a commercially available DNA extraction kit (QIAamp DNA Blood Mini-Kit, USA) according to the manufacturer's instructions. nPCR assays targeting the 18S ribosomal RNA (18S rRNA) gene were applied to detect piroplasm by using two pairs of primers (Piro1-S: 5′-CTTGACGGTAGGGTATTGGC-3′, Piro3-AS: 5′-CCTTCCTTTAAGTGATAAGGTTCAC-3′ and Piro-A: 5′-ATTACCCAATMCBGACACVGKG-3′ and Piro-B: 5′-TTAAATACGAATGCCCCCAAC-3′), as previously described [12,13]. The positive amplicons (∼400 bp) were purified by using a gel DNA purification kit (ZYMO, USA) according to the manufacturer's instructions. The purified amplicons were cloned into pGEM-Teasy vectors (Promega, USA) and recombinant plasmids were sent for sequencing to Genscript (Nanjing, China). The obtained sequences were run through a Blast analysis on the National Center for Biotechnology Information (NCBI) website using the BLASTn program. A set of primers, PIRO-F (5′-GGATAACCGTGSTAATTSTAGGGC-3′) and PIRO-R (5′-GTGTGTACAAAGGGCAGGGACG-3′), was used to amplify the long fragment of 18S rRNA for further confirmation of the *Babesia* species detection [13]. To avoid a risk of contamination, genomic DNA isolation from blood samples, PCR amplification and agarose gel electrophoresis were performed by different operators and in separated rooms. On the other hand, there was no human samples infected *Babesia* spp. including *B. divergens* in our laboratory based on our record.

Of the 754 blood samples, 10 (1.3%) were positive for piroplasms upon molecular amplification and gene sequencing. BLASTn results indicated that 10 sequences are identical to each other. A representative sequence was submitted to GenBank with accession number MK256977. These isolates could be classified as *B. divergens* group and shared 99.9% identity with that of *B. divergens* derived from Europe (Accession No. AY046576) (Figure 1). The age of the 10 patients ranged from 22 to 60 years (median 41.4 years old). All patients were immunocompetent and only two of them showed clinical symptoms at the time of sampling, such as fever, asthenia and headache. Clinical and laboratory data for the 10 patients are presented in Table 1.

**Figure 1.**
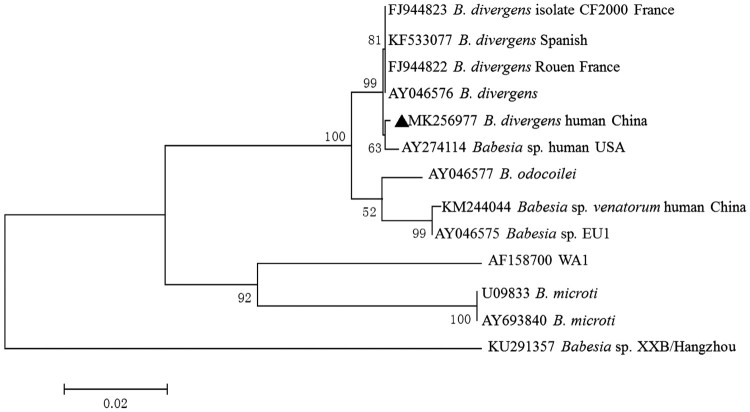
Phylogenetic tree of *Babesia* spp. sequences based on the 18S rRNA genes obtained in this study (bold triangles) and those previously registered in GenBank. The tree was constructed using the neighbour joining method of MEGA7, and values are given at the nodes. Numbers above the branch demonstrate bootstrap support from 1000 replications.

**Table 1.** Clinical information of 10 patients with *Babesia* species infections in Gansu province, China.

	Patient no.									
Characteristic	1	2	3	4	5	6	7	8	9	10
Sex	M	F	M	F	F	M	M	M	M	M
Age (year)	49	35	55	38	26	44	28	48	44	57
Clinical manifestation	–	–	–	–	–	Fever (38.5°C)	–	Asthenia	–	–
Leukocyte (10^9^/L)	5.6	5.6	12.5	13.2	17.7	14.4	7.9	9.6	11.5	7.2
Red blood cells (10^12^/L)	7.0	5.6	5.1	3.4	4.8	5.5	5.6	6.2	5.6	4.4
Haemoglobin concentration (g/L)	232	162	155	130	137	171	170	187	171	143
Platelet (10^9^/L)	107	253	280	319	293	240	274	230	216	166
Lymphocyte (10^9^/L)	1.7	3.0	1.7	1.3	2.0	3.9	3.3	2.6	2.7	2.5
Neutrophil (10^9^/L)	3.6	2.2	9.8	11.2	14.9	10.2	3.6	6.1	7.9	3.2
Hospital stay	NO	NO	Yes	Yes	Yes	Yes	NO	NO	NO	NO
*Babesia* species	Bdiv	Bdiv	Bdiv	Bdiv	Bdiv	Bdiv	Bdiv	Bdiv	Bdiv	Bdiv

–, No clinical manifestations of fever, headache, anorexia, myalgia, arthralgia, rash. Bdiv, *B. divergens.* Normal reference ranges: leukocyte count, 3.97–9.15 × 10^9^/L; red blood cells count, 4.09–5.74 × 10^12^/L; haemoglobin concentration, 131–172 g/L; platelet count, 85–303 × 10^9^/L; lymphocytes count, 0.8–4 × 10^9^/L; neutrophils count, 1.83–6.4 × 10^9^/L.

In this study, our findings have several unusual characteristics. First, several cases of *Babesia* infections in human were identified in Gansu province located in northwestern rather than eastern or northeastern China, where almost all of these cases were already reported. Our study is the first to report the presence of *Babesia* infection in humans in Gansu province, northwestern China. The infected parasites were identified as *B. divergens* that is an etiological agent of bovine babesiosis but has not been identified in cattle in China. By contrast, *B. divergens* is associated with poor patient outcomes, particularly in aged, asplenic and/or immunocompromised patients.

To our knowledge, only one case of human babesiosis, caused by *B. venatorum*, has been reported in northwestern China, in Xinjiang, with most occurrences (more than 100 cases) of babesiosis reported in eastern or northeastern China [11]. One case and eleven cases caused by *B. microti* were reported in Yunnan province (southwestern China) and Zhejiang province (eastern China), respectively. Two cases caused by *B. divergens* were reported in Shandong province in eastern China, whereas 48 cases caused by *B. venatorum* and 31 caused by *B. crassa* were reported in forested areas of northeastern China [9,13]. *Babesia* sp. XXB/HangZhou infection in an immunocompetent patient was reported in Zhejiang province in eastern China [8]. Additionally, the etiological agents of several human babesiosis cases, reported in Chongqing and Yunnan provinces, in China, have not been identified.

Most of the patients enrolled in this study visited hospital either for physical examination or as outpatients; therefore, it was not possible to collate data concerning the outcomes of infection for these patients. However, our results suggest that human babesiosis is prevalent in this region and, thus, residents should take steps to protect themselves against exposure to ticks. Asymptomatic patients carrying etiological agents of human babesiosis in their blood might be responsible for increasing the number of cases of transfusion-transmitted babesiosis [14,15]. Thus physicians should pay increased attention to this life-threating disease and be aware of the differential diagnoses and treatments available for babesiosis. Given the threat to public health, systematic epidemiological surveys should also be carried out to determine the real prevalence of this disease.
